# High Level of Nitrogen Makes Tomato Plants Releasing Less Volatiles and Attracting More *Bemisia tabaci* (Hemiptera: Aleyrodidae)

**DOI:** 10.3389/fpls.2017.00466

**Published:** 2017-03-31

**Authors:** Md. Nazrul Islam, Abu Tayeb Mohammad Hasanuzzaman, Zhan-Feng Zhang, Yi Zhang, Tong-Xian Liu

**Affiliations:** ^1^State Key Laboratory of Crop Stress Biology for Arid Areas, Northwest A&F UniversityYangling, China; ^2^Key Laboratory of Integrated Pest Management on the Loess Plateau of Ministry of Agriculture, Northwest A&F UniversityYangling, China; ^3^Agrochemical and Environmental Research Division, Institute of food and radiation Biology, Atomic Energy Research EstablishmentDhaka, Bangladesh; ^4^Vertebrate Pest Division, Bangladesh Agricultural Research InstituteGazipur, Bangladesh

**Keywords:** *Bemisia tabaci*, tomato plant, nitrogen, plant volatiles, SPME, GC-MS

## Abstract

Tomato (*Solanum lycopersicum*) production is seriously hampered by the infestation of the sweetpotato whitefly, *Bemisia tabaci* MEAM 1 (Middle East-Asia Minor 1). The infestation behavior of the whiteflies could be affected by the quantity of plant released volatile organic compounds (VOCs) related to nitrogen concentrations of the plant. In this study, we determined the infestation behavior of *B. tabaci* to the tomato plants that produced different levels of VOCs after application of different levels of nitrogen with a wind tunnel and an olfactometer. We also analyzed the VOCs released from nitrogen-treated tomato plants using solid phase microextraction and gas chromatography-mass spectrometry. The results revealed that the production of eight VOCs (β-pinene, (+)-4-carene, α-terpinene, p-cymene, β-phellandrene, α-copaene, β-caryophyllene, and α-humulene) was reduced after the plants were treated with high levels of nitrogen. However, more whiteflies were attracted to the tomato plants treated with high levels of nitrogen than to the plants treated with normal or below normal levels of nitrogen. These results clearly indicated that nitrogen can change the quality and quantity of tomato plant volatile chemicals, which play important roles in *B. tabaci* host plant selection.

## Introduction

Tomato, *Solanum lycopersicum* L., is an important vegetable in the world (Olaniyi et al., 2010; Bhowmik et al., 2012). There are many pests that cause both qualitative and quantitative losses of tomato (Lange and Bronson, 1981; Bari and Sarder, 1998). The sweetpotato whitefly, *Bemisia tabaci* (Genn.) (Hemiptera: Aleyrodoidea) is a major pest insect of vegetables, broadleaf field crops, and ornamentals in the tropics and sub-tropics of the world and in the protected environments of other areas (Liu, 2007). It is also considered one of the most important pests of tomato in the tropical and sub-tropical regions, causing heavy losses to crops by direct feeding and by transmitting geminiviruses (Toscano et al., 2002; Inbar and Gerling, 2008).

Many abiotic factors have been shown to influence the emission of VOCs that affect the host preference of insect pests, their colonization and life histories. Nitrogen is an abiotic factor that could affect the emission of volatiles from crop plants and further affect the behaviors of insects (Berenbaum, 1995; Duffey and Stout, 1996; Veromann et al., 2013; Han et al., 2014). As tomato is a high valued commercial crop, tomato growers have a tendency to over-use fertilizers (Locascio et al., 1992). Many studies have showed that excessive nitrogen application above the standard recommendation often increases nitrogen leaching, causing soil and water pollution, and increasing cost (Zotarelli et al., 2007; Sieling and Kage, 2010; Engström et al., 2011). Most plant feeding insects have a capability to search for the plants with high nitrogen content (Southwood, 1973). Although nitrogen fertilization rarely affects herbivores directly, it can change or alter morphological, physiological, and biochemical characters of host plants and increase food quality for herbivores (Bernays, 1990; Simpson and Simpson, 1990). In general, nitrogen content of a host plant is considered as an indicator of nutritional quality and a factor influencing host plant selection by plant feeding insects (Mattson, 1980). Several studies showed that nitrogen application modifies plant biochemical contents and pest resistance against herbivores. For example, Han et al. (2014) found that sub-optimal nitrogen supply is not favorable for the survival and development of the tomato leafminer *Tuta absoluta* (Meyrick), and this may be due to an increase of leaf chemical defense system and decrease in leaf nutritional value. In another experiment, Chen et al. (2008) found that cotton plants with high nitrogen were preferred for oviposition by the female beet armyworm *Spodoptera exigua* (Hübner). Moreover, Bentz et al. (1995a,b) found that protein-nitrogen content was linearly increased in the leaves of the poinsettia (*Euphorbia pulcherrima* Willd. et Kl.) plant with increasing level of nitrogen application, and *B. tabaci* host selection was also linearly increased with increasing of nitrogen content in the plants.

The composition and levels of plant VOCs might be significantly affected by nitrogen fertilization (Chen et al., 2010) and consequently affecting their attractiveness to pests. The relation between the levels of fertilizer application and the emission of plant volatiles depends on the plant species either there can be a positive, negative or no relation between them. For instance, Jang et al. (2008) observed a reduction in jasmonic acid levels in rice plants when receiving high amounts of nitrogen in all three cultivars tested. Van Wassenhove et al. (1990) also confirmed that constitutive volatile chemicals extracted from celery significantly decreased with increasing of high levels of mineral and/or organic nitrogen fertilizers. On the other hand, Gouinguenè and Turlings (2002) found that a lower quantity of volatile compounds was released from unfertilized corn plants (*Zea mays* L. var. *Delprim*) compared to those receiving a complete nutrient solution. Therefore, the quantitative differences of VOCs released from different plant species can vary when plants receive different levels of fertilization. However, the effects of nitrogen on the pattern of release of VOCs might be system- or species-specific, and there might be a correlation between nitrogen application, volatiles production, and host plant preference of insect pests.

Chemical communication between host plants and herbivores mostly depends on the herbivore and plant species. It is also based on multiple compounds of the plants (Blight et al., 1997). It is well known that many herbivorous insect pests choose their hosts based on visual modalities (optical characteristics of plants), semiochemical stimuli (plant volatile compounds), or both (Bernays and Chapman, 1994a; Schoonhoven et al., 2005; Cook et al., 2007a,b; Hasanuzzaman et al., 2016). In a study, Bleeker et al. (2009) found that monoterpenes and sesquiterpenes released from tomato plants stimulated a response from receptors on the antennae of *B. tabaci* and these terpene volatiles played an important role in a free-choice bioassay. Ying et al. (2003) also demonstrated that *B. tabaci* can distinguish different types of host plant volatiles without any visual references.

Host plant volatiles can act as repellents or attractants for herbivores (Unsicker et al., 2009; Dicke and Baldwin, 2010; Mumm and Dicke, 2010). The defensive and nutritional chemistry of the plant leaf is one of the factors that influences host choice and fitness of herbivore insects (Bernays and Chapman, 1994b). Fertilization and allelochemicals can influence selection of hosts by pest population (Slosser et al., 2004; Chau et al., 2005; Germano et al., 2011). In this study, we hypothesized that nitrogen fertilization levels affect the abundance of *B. tabaci*, and that this potential modification is related to changes in volatile compound bouquets of the plants. We tested our hypothesis by applying different doses of a nitrogenous fertilizer to tomato plant and observed host preferences of the whiteflies through olfactory responses, and determined the plant volatiles emitted from the treated tomato plants. The results of this study deliver evidences that the composition of the volatile compounds from tomato plant is associated with the nitrogen fertilization and this influences host plant selection by *B. tabaci*.

## Materials and Methods

### Plant Growth and Fertilization

Tomato plant [*Solanum lycopersicum* L., cv. ‘Gan Liang Mao Fen 802 F_1_’ seeds (Xian Qinshu Seeds Company Limited, Xian, Shaanxi, China)] were purchased from a local market. The seeds were sown in small plastic pots (7 cm × 7 cm × 8 cm) containing perlite as a plant-growing medium. The plants were grown inside insectaries where environmental conditions maintained at 25 ± 1°C with 60 ± 5% RH and 16:8 h L:D at a light intensity of 1400–1725 lux. All plants were nurtured in insect-proof mesh cages (60 cm × 60 cm × 60 cm).

We used four levels of nitrogen for growing tomato plants namely, 5 mM (T_1_), 10 mM (T_2_), 15 mM (T_3_), and 20 mM (T_4_) nitrogen where 5 mM was considered as a below normal level, 10 mM a normal level, and 15 mM and 20 mM were as a high level. Wahle and Masiunas (2003) and Wang et al. (2007) reported that the growth and yield of tomato was highest at near about 10 mM nitrogen levels but the tomato growth rate was limited with below 5 mM of nitrogen solution. Nutrient solutions for growing tomato plants were prepared with 5 mM nitrogen from a commercial fertilizer, ‘Kang Pu Jin’ with 20-20-20 of N-P_2_O_5_-K_2_O + Mg + TE (COMPO Expert GmbH, Krefeld, Germany), which was used as a standard control in all treatments. For other three treatments, i.e., T_2_, T_3,_ and T_4_, the source of additional nitrogen was urea (Sigma U 1250, purity > 99.5%). The nutrient combination of above mentioned fertilizer was N-P_2_O_5_-K_2_O (20%-20%-20%), Mg (0.3%) and other necessary trace elements, including S (0.8%), B (0.01%), chelated Cu (0.04%), chelated Fe (0.1%), chelated Mn (0.1%), chelated Zn (0.04%), and Mo (0.003%). The pH of the nutrient solutions was corrected at 6.0–6.5 if needed to add concentrated HCl or NaOH. The nutrient solutions were prepared with de-ionized water (Millipore, Rios^TM^ 5).

Different levels of nitrogen containing fertilizer solutions were applied to tomato plants following the technique of Stout et al. (1998) with slight modification. Tomato seeds were sown in the small plastic pots (7 cm × 7 cm × 8 cm) containing perlite as a plant-growing medium and watered with de-ionized water (Millipore, Rios^TM^ 5) up to 8 days after seed sowing. The plants were randomly assigned as T_1_, T_2_, T_3,_ and T_4_ treatments for 5, 10, 15, and 20 mM nitrogen, respectively. From 9th to 14th day, all plants were fertilized daily with 20 ml of 5 mM nitrogen prepared nutrient solution to avoid severe changes in the level of nitrogen applied to a plant. From days 15 to 20, all plants were fertilized daily with 20 ml of 10 mM nitrogen prepared nutrient solution except T_1_ treatment, which were fertilized daily with 5 mM nitrogen prepared nutrient solution. From days 21 to completion of the experiment, T_3_ and T_4_ treatments were fertilized daily with 20 ml of 15 mM and 20 mM nitrogen prepared nutrient solutions, respectively. At that time, T_1_ and T_2_ treatments were fertilized daily with 20 ml of 5 mM and 10 mM nitrogen prepared nutrient solutions, respectively, as followed in previous fertilization. To avoid water stress, additional de-ionized water was added as necessary. Thirty-five days old intact tomato plants were used for all experiments, i.e., wind tunnel and Y-tube bioassays, determination of total nitrogen and volatile compounds containing four to five fully expanded leaves (Supplementary Figure S1).

### Insect Rearing

The whiteflies, *B. tabaci* MEAM1 (Frohlich et al., 1999), were mass-reared in walk-in insectaries in large screen cages (60 cm × 60 cm × 60 cm) on eggplant *Solanum melongena* L. (Solanaceae) cv. ‘Zichangqie’ which was grown in 15 cm diameter plastic pots containing 5:1:1 by volume of peat moss, perlite and vermiculite. The insectaries were maintained at 25 ± 1°C with 60 ± 5% RH and a 16:8 h L:D at a light intensity of 1400–1725 lux, which was similar to the experimental environments in which tomato plants were grown.

### Wind Tunnel Bioassays

Response of adult *B. tabaci* toward different levels of nitrogen applied tomato plants was tested in a plexiglass wind tunnel (200 cm × 70 cm × 70 cm) which was set up in a small controlled environmental room (temperature 25 ± 1°C, RH 60 ± 5%). A blower pulled the air, and the airflow rate was adjusted at 22 cm s^−1^. Three 18 W fluorescent lamps were set up at the take-off point in the flight chamber. The plastic pot except the plant was covered with polythene bags (EasyOne Oven Bags, Reynolds Kitchens, Lake Forest, Illinois, USA) and aluminum foil to minimize plant-growing media produced volatiles before using the plants. In this test, at a time two different nitrogen doses applied intact tomato plants were placed next to each other on the upwind end of the arena maintaining 30 cm distance to observe that the male or female *B. tabaci* would fly upwind in the presence of both visual and volatile plant cues. A petri dish containing 100 male or female *B. tabaci* was kept inside the wind tunnel maintaining a short-distance (1-m) from the two plants. Thereafter, *B. tabaci* were released there to make a choice their suitable host. The *B. tabaci* were released at 12:00 h, and 24 h later, the individuals were carefully counted to investigate their preference between two nitrogen treatments in the presence of both visual and olfactory cues. Numbers of whitefly adults were counted 24 h later because it took the adults some time to make a choice and settle down in the wind tunnel against wind flow. Before mass releasing of the whiteflies, five individuals were pre-released to ensure that the mass release does not affect the responses of the whiteflies to the odor sources. Each experiment was conducted three times. The wind tunnel was wiped thoroughly with 70% ethanol and air was pulled through the wind tunnel for at least 2 h before it was re-used.

### Olfactory Choice Test with Y-Tube Olfactometer

A Y-tube olfactometer was used to evaluate the behavioral response of *B. tabaci* to nitrogen applied tomato plant volatile compounds, following the procedure as previously described by Akol et al. (2003) and Saad et al. (2015) with some modifications. The transparent glass made Y-tube olfactometer consisted of 8 cm long base with 0.8 cm internal diameter, two 8 cm lateral branches at a 60° angle from each other. The lateral branches were individually connected to two 3 L glass container with odorless tubes and each glass container contained volatiles releasing one intact tomato plant from each nitrogen treatments. The plant was kept in the glass container for 1 h before sampling for exiting impure volatiles from the system. Plant containing glass containers were covered with thick paper so that *B. tabaci* individual cannot receive visual cues from the plants. Charcoal filtered humidified and purified air was provided at 100 ml min^−1^ to both branches of the Y-tube via odor sources using a vacuum pump (Beijing BCHP Analytical Technology Institute, China) for circulating the volatile organic compounds. The air flow was adjusted and measured by an inline flowmeter (LZB-3WB, Changzhou, Jiangsu, China). Male and female *B. tabaci* were tested separately. Adult whiteflies were starved for 2 h and then released within 0.5–1.0 cm of the base of the Y-tube with a small PCR tube and their responses assessed for 10 min. A *B. tabaci* adult that walked into one of the lateral branches of the Y-tube at least 5 cm, stayed a minimum of half a minute and did not return was considered as a positive, responsive individual. If an individual did not make a decision within 10 min, they were excluded from the results, and considered as non-responsive insect. To eliminate lighting bias, a 20 W fluorescent light was placed vertically 0.5 m over the Y-tube olfactometer. The positions of two lateral branches of the Y-tube were inverted 180° after every five insects tested. Each olfactory test was repeated two times for each combination of stimulus pairs, and each replicate consisted of 25 *B. tabaci* adults tested individually, i.e., a total of 50 *B. tabaci* adults for each treatment were assayed. The experiment was carried out between 12.00 and 16.00 h in a controlled environment maintained at 25 ± 1°C and 60 ± 5% relative humidity. To minimize plant-growing media produced volatiles, the pot except the plant was covered with the same polythene bags and aluminum foil as described previously. The experimental Y-tube and all glassware were washed with soap with tap water, then distilled water, and finally sterilized with 70% ethyl alcohol before oven drying (120°C for 3 h) to reduce the contamination risk of previous tested odors.

### Determination of Nitrogen from Tomato Plants

Tomato plants (leaves with stem) were taken separately from each treatment and oven dried at 65°C for 4 days. The dried plant samples were ground with a mortar and a pestle, taken in poly bag and preserved it at room temperature before analysis. Total nitrogen from tomato plant tissues was measured using the Kjeldahl method. Exactly 0.1 g sample was digested with 4 ml of H_2_SO_4_ at 420°C for 1 h along with catalyst of K_2_SO_4_ and CuSO_4_ at a ratio of 9:1. FOSS 8400 automatic Kjeldahl apparatus (FOSS Analytical AB, Sweden) was used to analyze total nitrogen from tomato plant tissues. Five samples from each treatment were used to measure the quantity of nitrogen.

### Volatile Compound Collection from Nitrogen Applied Tomato Plants

**FIGURE 1:**
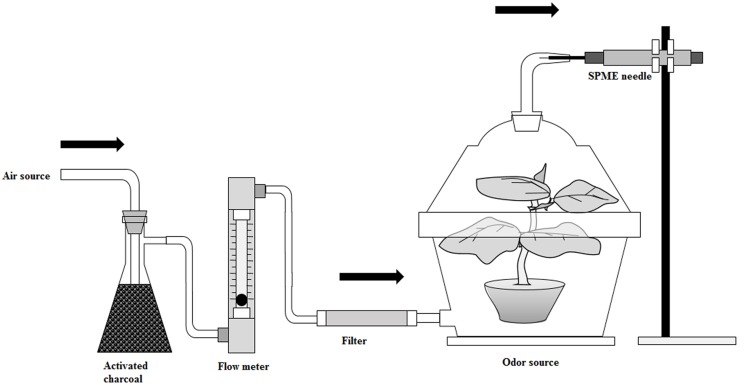
**A schematic diagram of volatile organic compound collection**.

Different levels of nitrogen-treated tomato plants released dynamic headspace volatiles were collected with solid phase micro extraction (SPME) fiber coated with poly dimethyl siloxane-divinyl benzene (PDMS-DVB, 65 μm) purchased from Supelco (Bellefonte, PA, USA). The volatile collection procedure is shown in **Figure 1**. Previously, many researchers collected VOCs in headspace static using the SPME method by placing a plant in a closed chamber, and this method consequently increased the temperature and relative humidity in the chamber, which could affect the normal physiological process in volatile chemicals emissions (Gouinguenè and Turlings, 2002; Egigu et al., 2014). In our experiment, VOCs were collected using the headspace dynamic SPME method. Here, a continuous airflow went through a 35-days old plant which was confined inside a glass jar (3 L) that minimized the variation of temperature and humidity inside the glass jar. Again, the same polythene bags and aluminum foil were used to avoid plant-growing media produced volatiles as described previously. On the top of the lid of the glass container was a large hole that closed with a glass made cover containing a bend opening (4 mm in diameter) which was used to insert the SPME fiber. At the bottom of the glass container was another hole for ventilation. A vacuum pump was used for circulating the air which was purified with activated charcoal and thereafter a Tenax TA adsorbent. The flow rate of the air was maintained at 200 ml min^−1^ with a flow meter. The SPME fiber was conditioned at 250°C for 30 min in a gas chromatograph injection port according to the guideline of the manufacturer. The plant was kept in the glass container for 1 h before sampling for exiting impure volatiles from the system. The SPME needle was inserted into the opening of the glass container and extended the fiber to absorb dynamic headspace plant volatiles which escaped through the opening of the glass container for 1 h. After absorption of the volatiles, the SPME needle was directly inserted into a gas chromatograph-mass spectrometer (GC-MS) thermal desorption port as soon as possible, and thereafter the fiber was extended and kept 5 min for desorption of volatile substances.

### Analysis of Volatile Compounds

The system consisted of a GC (TRACE 1310, Thermo Fisher Scientific, Waltham, MA, USA) that was used for the separation of volatile chemicals and an MS (ISQ Single Quadrupole MS, Thermo Fisher Scientific, Waltham, MA, USA) used for their detection, identification, and quantification. The thermally desorbed VOCs were separated in a 30 m × 0.25 mm × 0.25 μm film thickness fused silica capillary column (Zebron, ZB-5 MS Ui). Programming splitless injector temperature was maintained at 250°C whereas MS transfer line and ion source temperatures were maintained at 280°C. The purified helium (99.999%) was used as a carrier gas and the flow rate was maintained at 1.0 ml min^−1^ with constant mode. The initial GC oven temperature was set to 40°C for 4 min. The oven temperature was increased from 40 to 250°C at a rate of 8°C min^−1^ and held for 5 min. The MS was operated in an electron ionization (EI) mode. The ion energy and emission current were maintained at 70 eV and 25 μA, respectively. The Xcalibur program (Ver. 2.1, Thermo Electron Corporation, USA) was used for data acquisition which was performed in a total ion chromatogram (TIC) with mass range from 33 to 500 amu. The identification of separated compounds was carried out with NIST 2008 (National Institute of Standards and Technology, Washington, DC, USA) database. Kovats retention index (KI) was calculated for each constituent in relation to a mixture of *n*-alkanes standard (Van Den Dool and Kratz, 1963), and C_7_–C_40_ (Sigma–Aldrich, Louis, MO, USA). The data were matched to previously published data (**Table 1**). The peak area of each component of the volatiles was the relative quantity (Fang et al., 2013).

**Table 1:** Molecular weight, mass peak (m/z), retention time (RT), calculated Kovats indicies (CKI) and tabulated Kovats retention indices (TKI) of the volatile compounds identified from intact tomato plants after four nitrogen treatments (see **Figure 6** for relative amounts of volatile organic compounds indicated by peak areas found from different levels of nitrogen-treated tomato plants).

Peak no	Compounds	Chemical class	Molecular weight	Mass peak (m/z)	RT (min)	CKI	TKI	Reference
1	Heptanal	Alkyl aldehyde	114	70, 44, 41, 55	6.95	903	901	Babushok and Zenkevich, 2008
2	α-Pinene	Monoterpene	136	93, 91, 92, 77	7.65	932	932	Adams, 2009
3	β-Pinene	Monoterpene	136	93, 41, 69, 91	8.58	970	973	Franco et al., 2011
4	Myrcene	Monoterpene	136	41, 93, 69, 53	9.14	993	990	Adams, 2009
5	(+)-4-carene	Monoterpene	136	93, 121, 136, 91	9.30	1000	1001	Le Quere and Latrasse, 1990
6	α-Terpinene	Monoterpene	136	93, 121, 91, 136	9.69	1018	1019	Franco et al., 2011
7	p-Cymene	Monoterpene	134	119, 134, 91, 120	9.86	1025	1023	Franco et al., 2011
8	β-Phellandrene	Monoterpene	136	93, 77, 91, 136	9.94	1029	1029	Franco et al., 2011
9	Nonanal	Alkyl aldehyde	142	57, 41, 43, 56	11.63	1108	1108	Engel et al., 2002
10	δ-Elemene	Sesquiterpene	204	121, 93, 41, 107	15.96	1343	1338	Adams, 2009
11	α-Copaene	Sesquiterpene	204	161, 119, 105, 93	16.61	1382	1376	Franco et al., 2011
12	Longifolene	Sesquiterpene	204	161, 94, 91, 93	17.12	1413	1416	Hognadottir and Rouseff, 2003
13	α-Cedrene	Sesquiterpene	204	119, 93, 105, 204	17.23	1420	1419	Da Cruz et al., 2014
14	β-Caryophyllene	Sesquiterpene	204	93, 133, 91, 41	17.34	1427	1425	Da Cruz et al., 2014
15	α-Humulene	Sesquiterpene	204	93, 80, 41, 121	17.88	1462	1460	Da Cruz et al., 2014
16	Farnesan	Alkane	212	57, 71, 43, 41	20.01	1604	–	–

### Statistical Analyses

IBM SPSS statistics version 19 (Chicago, IL, USA) was used to conduct all statistical analyses. Data produced from Y-tube olfactometer choice bioassays were analyzed by **X**^2^ test. Wind tunnel bioassay data were analyzed by paired *t*-test. Tomato plant volatiles and nitrogen were analyzed through one-way analysis of variance (ANOVA); means were separated by the Tukey test. Correlation was used to identify a possible relationship between different levels of nitrogen and volatile constituent production of tomato plants. In all cases, means were considered significant at *P* < 0.05 level.

## Results

### Two-Way Choice Tests Conducted in a Wind Tunnel

**FIGURE 2:**
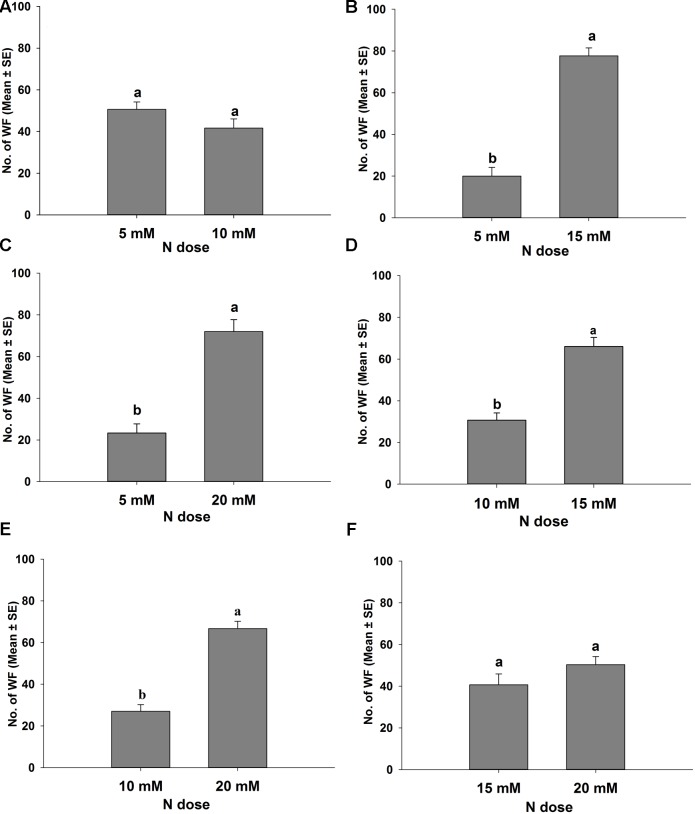
**Response of *Bemisia tabaci* females in different levels of nitrogen applied tomato plants in a wind tunnel experiment.** Different letters are denoted significantly different at *P* < 0.05, and the same letters are denoted not-significant at *P* = 0.05 (Paired *t*-test). **(A)** 5 and 10 mM N; **(B)** 5 and 15 mM N; **(C)** 5 and 20 mM N; **(D)** 10 and 15 mM N; **(E)** 10 and 20 mM N; **(F)** 15 and 20 mM N treated tomato plants.

**FIGURE 3:**
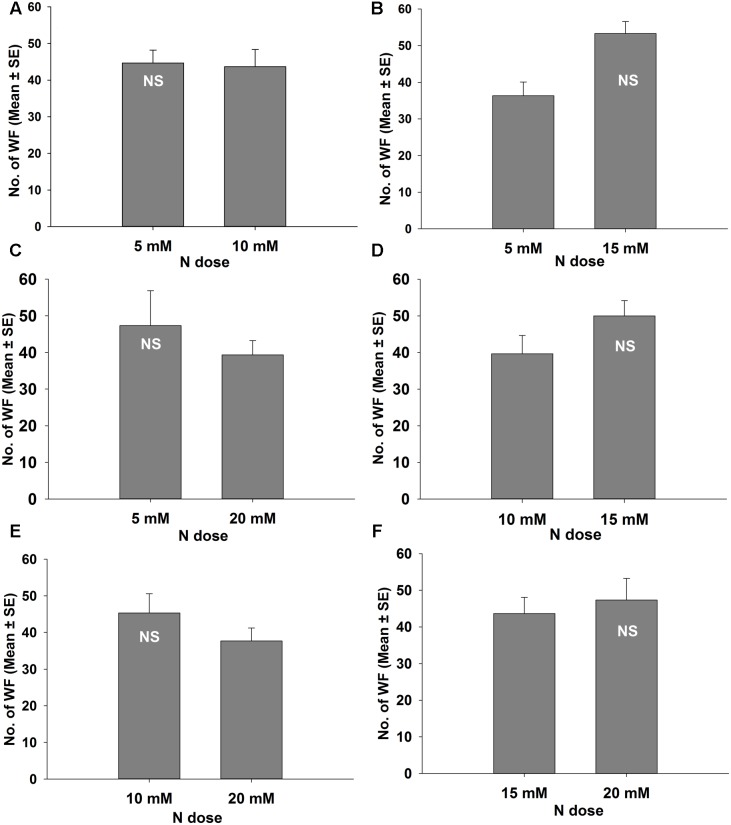
**Response of *B. tabaci* males in different levels of nitrogen applied tomato plants in a wind tunnel experiment.** NS, denoted not significant at *P* = 0.05 (Paired *t*-test). **(A)** 5 and 10 mM N; **(B)** 5 and 15 mM N; **(C)** 5 and 20 mM N; **(D)** 10 and 15 mM N; **(E)** 10 and 20 mM N; **(F)** 15 and 20 mM N treated tomato plants.

Different levels of nitrogen-treated tomato plants were tested in the wind tunnel to investigate their attractiveness to *B. tabaci* in the presence of both visual and olfactory cues. The results of these dual choice bioassays are presented in **Figure 2** for *B. tabaci* females and **Figure 3** for *B. tabaci* males. The attractiveness of *B. tabaci* females was found different in 5, 10, 15, and 20 mM N treated tomato plants. The results revealed that significantly more *B. tabaci* females were found in 15 mM N (*t* = 7.317, *P* < 0.05; **Figure 2B**) and 20 mM N (*t* = 5.034, *P* < 0.05; **Figure 2C**) than in 5 mM N treated tomato plants. When given an option to choose 10 mM N versus 15 mM N treated plants_,_ *B. tabaci* females preferred 15 mM N to 10 mM N treated plant (*t* = 4.508, *P* < 0.05; **Figure 2D**). When 10 mM N and 20 mM N were offered, *B. tabaci* females preferred 20 mM N to 10 mM N treated plant (*t* = 6.263, *P* < 0.05; **Figure 2E**). However, *B. tabaci* females did not show significant preference between 5 mM N and 10 mM N (*t* = 1.199, *P* = 0.353; **Figure 2A**) and between 15 mM N and 20 mM N treated tomato plants (*t* = 1.609, *P* = 0.249; **Figure 2F**).

The data from the wind tunnel dual choice test showed that adult *B. tabaci* males did not have significant preference between 5 mM N and 10 mM N (*t* = 0.122, *P* = 0.914; **Figure 3A**), between 5 mM N and 15 mM N (*t* = 2.592, *P* = 0.122; **Figure 3B**), between 5 mM N and 20 mM N (*t* = 0.610, *P* = 0.604; **Figure 3C**), between 10 mM N and 15 mM N (*t* = 1.131, *P* = 0.375; **Figure 3D**), between 10 mM N and 20 mM N (*t* = 1.113, *P* = 0.382; **Figure 3E**), and between 15 mM N and 20 mM N (*t* = 0.355, *P* = 0.757; **Figure 3F**) treated tomato plants.

### Olfactory Bioassay with Y-Tube Olfactometer

**FIGURE 4:**
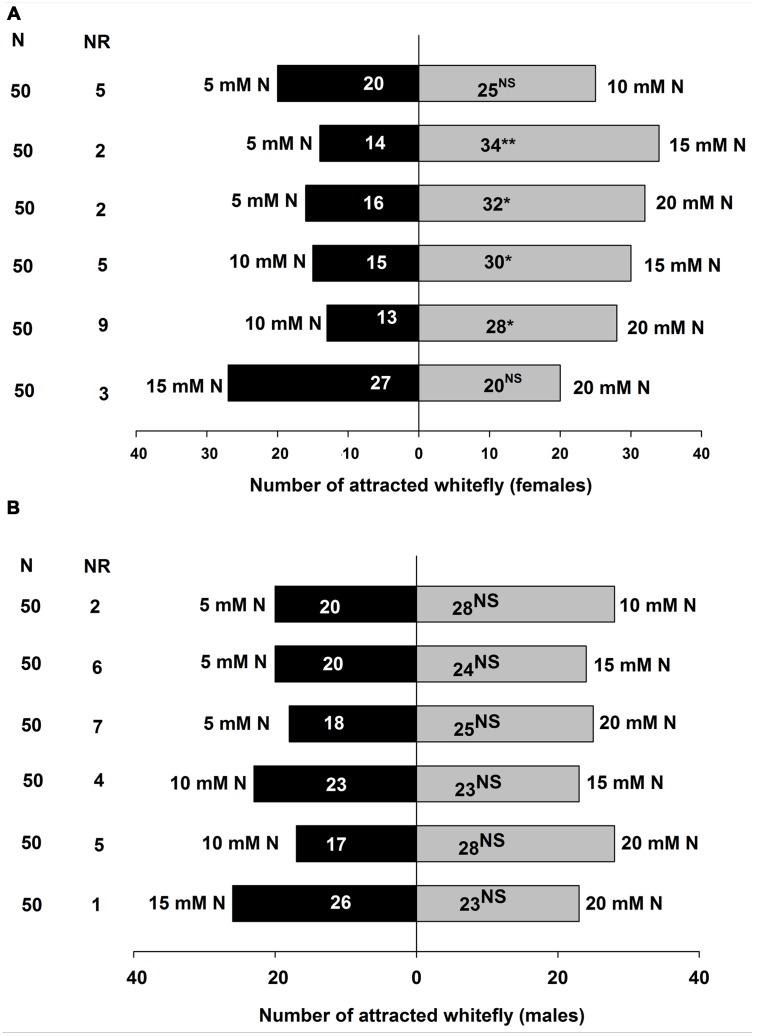
**Olfactory responses of *B. tabaci* females (A)** and males **(B)** in Y-tube olfactometer assay given a choice between volatile compounds from tomato plants treated with different doses of nitrogen. N, numbers of *B. tabaci* females and males tested. NR, Number of non-responsive insects. NS, Not significantly different at *P* = 0.05. ^∗^, Significantly different at *P* < 0.05. ^∗∗^, Significantly different at *P* < 0.01 (*X*^2^ test).

The preferences of *B. tabaci* were assayed in the Y-tube olfactometer toward volatile blends released from different levels of nitrogen-treated tomato plants, and the results are shown in **Figure 4A** for *B. tabaci* females and **Figure 4B** for *B. tabaci* males. Adult *B. tabaci* females showed significant preference among the VOCs of the plants treated with various levels of nitrogen including 5, 10, 15, and 20 mM N. Significantly more *B. tabaci* females were attracted to 15 mM N (χ^2^ = 8.333; *P* < 0.01) than 5 mM N when 5 mM N and 15 mM N were offered, and to 20 mM N (χ^2^ = 5.333; *P* < 0.05) when 5 mM N and 20 mM N were offered. Similarly, *B. tabaci* females preferred 15 mM N when 10 mM N and 15 mM N treated tomato plants were offered (χ^2^ = 5.000; *P* < 0.05), and preferred 20 mM N (χ^2^ = 5.488; *P* < 0.05) when 10 mM N and 20 mM N treated tomato plants were offered. However, *B. tabaci* females did not show significant preference between 5 mM N and 10 mM N (χ^2^= 0.556; *P* = 0.456), and between 15 mM N and 20 mM N (χ^2^ = 1.043; *P* = 0.307) treated tomato plants (**Figure 4A**).

However, in the choice tests, *B. tabaci* male adults did not show any significant preference among the VOCs from the plants treated with different levels of nitrogen (5 mM N and 10 mM N: χ^2^ = 1.333; *P* = 0.248; 5 mM N and 15 mM N: χ^2^ = 0.364; *P* = 0.546; 5 mM N and 20 mM N: χ^2^ = 1.140; *P* = 0.286; 10 mM N and 15 mM N: χ^2^ = 0.000; *P* = 1.000; 10 mM N and 20 mM N: χ^2^ = 2.689; *P* = 0.101; and 15 mM N and 20 mM N: χ^2^ = 0.184; *P* = 0.668) (**Figure 4B**).

### Amounts of Nitrogen in Tomato Plants

**FIGURE 5:**
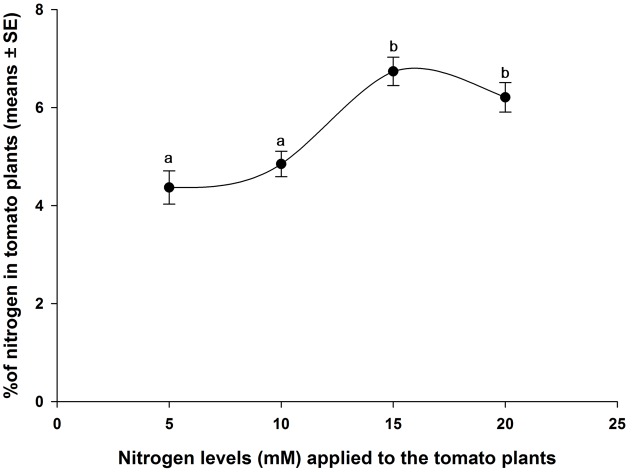
**Percentages of total nitrogen (means ± SE, *n* = 5) in tomato plant tissues.** The means in the figure with the same letters are not significantly different at *P <* 0.05 (Tukey test; one-way ANOVA).

The results of total nitrogen in different levels of nitrogen-treated tomato plants are shown in **Figure 5**. Significant variation of total nitrogen in tomato plants (leaves with stem) were found due to different levels of nitrogen application. Tomato plants grown in 15 mM (T_3_) and 20 mM (T_4_) nitrogen levels had significantly higher percentage of total nitrogen than the plants grown in 5 mM (T_1_) and 10 mM (T_2_) nitrogen (*F*_3,16_ = 12.163, *P* < 0.001). There was no significantly difference of percentages of total nitrogen in T_1_ and T_2_ treatments. Similarly, no significant difference of percentages of total nitrogen was observed between T_3_ and T_4_ treatments (**Figure 5**). However, different levels of nitrogen affected plant weight (Supplementary Figure S2).

### Volatile Organic Compounds Identified from Intact Tomato Plants after Application of Different Levels of Nitrogen, Quantity of Volatile Constituents and Correlation between Nitrogen Levels

**FIGURE 6:**
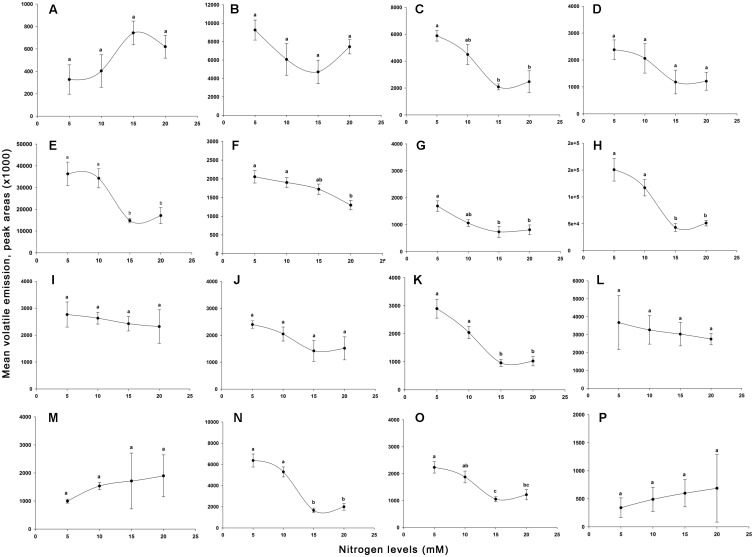
**Relative amounts of volatile organic compounds indicated by peak areas found from different levels of nitrogen-treated tomato plants.** Indicated peak areas (means ± SE, *n* = 4) found from g^−1^ fresh shoot weight h^−1^. Different capital letters are denoted the following volatiles. **(A)**, heptanal; **(B)**, α-pinene; **(C)**, β-pinene; **(D)**, myrcene; **(E)**, (+)-4-carene; **(F)**, α-terpinene; **(G)**, p-cymene; **(H)**, β-phellandrene; **(I)**, nonanal; **(J)**, δ-elemene; **(K)**, α-copaene; **(L)**, longifolene; **(M)**, α-cedrene; **(N)**, β-caryophyllene; **(O)**, α-humulene; **(P)**, farnesan. The means in the figure with the same small letters are not significantly different at *P* = 0.05 (Tukey test; one-way ANOVA)

**FIGURE 7:**
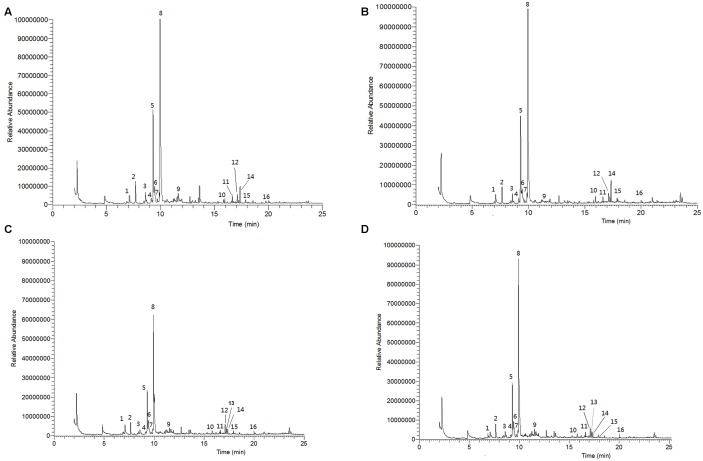
**Gas chromatography-mass spectrometry (GC-MS) total ion chromatograms (TIC) of volatile organic compounds emitted by different levels of nitrogen-treated tomato plants** (**A**, 5 mM; **B**, 10 mM; **C**, 15 mM, **D**, 20 mM of nitrogen-treated plants). Numbered emission peaks indicate the following volatiles. 1, heptanal; 2, α-pinene; 3, β-pinene; 4, myrcene; 5, (+)-4-carene; 6, α-terpinene; 7, p-cymene; 8, β-phellandrene; 9, nonanal; 10, δ-elemene; 11, α-copaene; 12, longifolene; 13, α-cedrene; 14, β-caryophyllene; 15, α-humulene; 16, farnesan.

Effects of nitrogen on tomato plant VOCs are shown in **Figures 6A–P**, and GC-MS chromatograms of volatiles are presented in **Figures 7A–D**. Sixteen VOCs were identified from the tomato plants with nitrogen fertilization treatments. Besides plant emitted VOCs, some compounds are generally related with the analytical system such as phthalates or siloxanes, as well as compounds related with earth’s atmosphere such as benzene and toluene (Warneke et al., 2001; Jansen et al., 2008) were not included in the list. The quantities of eight VOCs emitted from tomato plants, including heptanal (*F*_3,12_ = 2.461, *P* = 0.113; **Figure 6A**), α-pinene (*F*_3,12_ = 2.367, *P* = 0.122; **Figure 6B**), myrcene (*F*_3,12_ = 1.993, *P* = 0.169; **Figure 6D**), nonanal (*F*_3,12_ = 0.220, *P* = 0.881; **Figure 6I**), δ-elemene (*F*_3,12_ = 2.007, *P* = 0.167; **Figure 6J**), longifolene (*F*_3,12_ = 0.177, *P* = 0.910; **Figure 6L**), α-cedrene (*F*_3,12_ = 0.733, *P* = 0.552; **Figure 6M**), and farnesan (*F*_3,12_ = 0.178, *P* = 0.909; **Figure 6P**), were not significantly varied with increasing nitrogen treatments. The quantities in the remaining eight identified volatiles differed significantly among the N levels applied, including β-pinene (*F*_3,12_ = 8.863, *P* < 0.01; **Figure 6C**), (+)-4-carene (*F*_3,12_ = 7.853, *P* < 0.01; **Figure 6E**), α-terpinene (*F*_3,12_ = 5.290, *P* < 0.05; **Figure 6F**), p-cymene (*F*_3,12_ = 5.875, *P* < 0.05; **Figure 6G**), β-phellandrene (*F*_3,12_ = 14.110, *P* < 0.001; **Figure 6H**), α-copaene (*F*_3,12_ = 16.683, *P* < 0.001; **Figure 6K**), β-caryophyllene (*F*_3,12_ = 29.783, *P* < 0.001; **Figure 6N**), and α-humulene (*F*_3,12_ = 9.029, *P* < 0.01; **Figure 6O**). Of the eight significantly varied VOCs including β-pinene, (+)-4-carene, α-terpinene, p-cymene, β-phellandrene, α-copaene, β-caryophyllene, and α-humulene, no significant differences were found between 5 mM (T_1_) and 10 mM (T_2_) nitrogen-treated plant volatiles except for β-pinene, p-cymene, and α-humulene. The tomato plants at 15 mM (T_3_) and 20 mM (T_4_) nitrogen produced significantly lower levels of β-pinene, (+)-4-carene, α-terpinene, p-cymene, β-phellandrene, α-copaene, β-caryophyllene, and α-humulene as compared with the tomato plants grown at the 5 mM (T_1_) and 10 mM (T_2_) nitrogen. Of the eight significantly varied VOCs, no significant differences were observed between 15 mM (T_3_) and 20 mM (T_4_) nitrogen-treated plant volatiles except for α-terpinene and α-humulene. The amounts of significantly varied eight VOCs identified are well and negatively correlated with the N levels in the tomato plants, the higher the N levels, the lower of the VOCs identified (*r* = −0.883 to −0.967).

## Discussion

In our study, the tomato plants treated with below normal and normal nitrogen levels of nitrogen (T_1_ and T_2_) had similar plant nitrogen contents. Similarly, the tomato plants treated with higher nitrogen levels (T_3_ and T_4_) showed similar plant nitrogen contents (**Figure 5**). Han et al. (2014) reported that optimal nitrogen-treated tomato plants showed statistically similar leaf nitrogen content to those treated with high nitrogen, but the amount of tomato leaf nitrogen was statistically higher than the plants treated with low levels of nitrogen input. However, our results demonstrated that the nitrogen induced VOCs emitted from tomato plants influenced the behavioral responses of *B. tabaci* female to host plants. For instance, in the wind tunnel bioassay, *B. tabaci* females preferred higher levels of nitrogen-treated tomato plants (**Figure 2**). The Y-tube olfactometer tests were also provided strong evidence that *B. tabaci* females chose the VOCs from T_3_ and T_4_ plants without visual or physical contact with the plants (**Figure 4A**). In the free-choice experiment, the whiteflies could make a decision to choose their host plant with different morphological characteristics, volatile constituents of the plants, or both. However, in the olfactometer experiments, the whiteflies chose the plants only based on the VOCs from the plant, not the morphological characters. Addesso and McAuslane (2009) reported that without volatile compounds, the pepper weevil (*Anthonomus eugenii*) adults were more inclined to move downwind or remain stationary than to move upwind in the wind tunnel. Therefore, volatile compounds play a critical role to choose the host plant. In our study, the whiteflies showed similar preferences to the VOC from the plants treated with different amounts of nitrogen in both the wind tunnel and the olfactometer experiments. These results are consistent with the findings of Saad et al. (2015) who found that the host plant preference of *B. tabaci* was similar in the free-choice and olfactometer experiments.

The wind tunnel and Y-tube olfactometer bioassays revealed that the females and males of *B. tabaci* had different responses to the VOCs emitted from the plants treated with different amounts of nitrogen. For instance, the male adults of *B. tabaci* did not show any preference to the VOCs emitted from the plants treated with different amounts of nitrogen (**Figures 3**, **4B**). It is generally believed that females play a greater role in finding plants for oviposition for offspring development than males (Zu Dohna, 2006). Moreover, this host finding behavioral difference between the sexes supported the general pattern where many female insects are more attracted by host plant odors than males (Raguso et al., 1996; Zhang et al., 1999; Das et al., 2007; Szendrei and Rodriguèz-Saona, 2010). Saad et al. (2013) and Zheng et al. (2013) reported that *B. tabaci* and *Aleurodicus dispersus* females significantly preferred chili plant odors and hexanol isomers, respectively, in an olfactometer assay whereas the males did not show significant responses. Sex-specific olfactory responses and host selection of *B. tabaci* based on volatile compounds are comparatively poorly studied. It is possible that volatile chemicals emitted by host plants are assumed to mainly affect females (Finch, 1980), whereas pheromones stimulates higher responses from male insects than from female insects (Li and Maschwitz, 1985). Therefore, further studies on the differences between females and males of *B. tabaci* are needed.

The results of our wind tunnel and olfactometer experiments showed that higher amounts of nitrogen receiving plants, for example T_3_ and T_4_, were highly preferred by *B. tabaci* females compare to T_1_ and T_2_ nitrogen-treated plants, indicating that the behavioral response of *B. tabaci* females could be attributed quantitative differences of nitrogen induced volatile compounds released by the plants. In the volatile analysis test, we found that highly attractive plants emitted significantly less amount of monoterpenes (β-pinene, (+)-4-carene, α-terpinene, β-phellandrene, and p-cymene) and sesquiterpenes (α-copaene, α-humulene, and β-caryophyllene) from the intact tomato plants. This result indicated that production of monoterpenes and sesquiterpenes, generally decreased with the increase of nitrogen levels in tomato plants (**Figures 5**, **6**), and these terpene volatiles could influence the behavioral response of *B. tabaci*. This result is in agreement with those reported by Tuomi et al. (1994); Lee et al. (2005), and Chen et al. (2008), who found that plants grown at a high level of fertilizer had a lower terpene concentration than the plants grown in a low level of fertilizer. Similarly, Fretz (1976) reported that a low level of terpene was found in *Juniperus horizontalis* after additional nitrogen fertilizer application. It has been found that host plant resistance of *B. tabaci* is related to the optimal nutrient (nitrogen) content of the plants. Reddy and Rao (1989) and Bentz et al. (1995b) reported that nutrient levels (nitrogen) supplied to a plant can increase its nutrient quality, such as, increase of leaf nitrogen content could interfere the natural resistance mechanism of the host plant to insects. Nitrogen may influence nutritional values and semiochemicals of plants and also behavioral characteristics of herbivores. For instance, Jauset et al. (1995) and Zaini et al. (2013) showed that *B. tabaci* populations were higher when crops were provided with higher levels of nutrients than low levels of nutrients. Our results also showed that *B. tabaci* female preferred the plants with high levels of nitrogen. Therefore, we think that high levels of nitrogen-treated tomato plants exhibited different plant defense mechanism that become more attractive to *B. tabaci*.

The amount of plant VOCs released by individual plants can vary with abiotic factors that impact plant’s physiology. These variations may induce different defense mechanisms of the plants to herbivores, and further affect the infestation performance and behavior of insect pests (Gonzales et al., 2002). In our study, the tomato plant treated with normal and below normal levels of nitrogen emitted high amounts of volatile monoterpenes and sesquiterpenes compared to the plants treated high levels of nitrogen. Monoterpenes and sesquiterpenes increased with the decrease of nitrogen level in tomato plants are consistent with the carbon-nutrient balance hypothesis where predicted secondary metabolites production will be affected in case of lack of any nutrients (Gershenzon and Croteau, 1991). Growth rate of the plant will be reduced due to low nutrient availability but carbohydrate accumulation continues by constant photosynthesis due to carbon availability. Subsequently, accumulation of carbohydrate will lead to synthesis of constitutive secondary compounds like terpenoids (Herms and Mattson, 1992; Gershenzon, 1994). Therefore, we think that T_1_ and T_2_ nitrogen-treated tomato plants produced high amount of insect repelling terpenes, especially, monoterpenes and sesquiterpenes than T_3_ and T_4_ nitrogen-treated tomato plants, which is the possibly reason to move a higher number of *B. tabaci* females to T_3_ and T_4_ nitrogen-treated tomato plants in both wind tunnel and olfactometer experiments. These terpene volatiles released from plants in low amount are often reduced in defense against herbivores (Bleeker et al., 2009; Fang et al., 2013). A number of monoterpenes and sesquiterpenes have been reported to display repellent property to *B. tabaci*. For example, virus infected tomato plant was significantly susceptible to *B. tabaci* and that plant had a significant lower concentration of the volatiles α-pinene, limonene, 4-carene, thymine, β-phellandrene, caryophyllene, α-cedrene, β-cedrene, and α-humulene than the healthy plant (Fang et al., 2013; Luan et al., 2013). Similarly, significantly less amount of monoterpenes (e.g., p-cymene, 1, 8-cineole) and sesquiterpenes (e.g., α-copaene, β-cedrene) emitting healthy plants were more preferred by *B. tabaci* females than infested plants (Saad et al., 2015). Our results also showed that the concentration of certain terpenes decreased with the increase of nitrogen levels in tomato plants that was significantly attractive to *B. tabaci* as shown in the wind tunnel and olfactometer experiments. Zhang et al. (2004) and Yang et al. (2010) conducted a similar olfactory bioassay of *B. tabaci* with a vertical olfactometer and showed that ginger oil extract repelled adult *B. tabaci*. A mixture of volatile constituents, including monoterpenes (p-cymene, α-terpenene, β-phellandrene, 1,8-cineole, camphene), sesquiterpenes, alcohols, and aldehydes, were associated to the repellent properties of an essential oil (Owolabi et al., 2007; Ukeh et al., 2009; Sa-Nguanpuag et al., 2011; Nampoothiri et al., 2012). Similarly, Simmons and Gurr (2005) and Bleeker et al. (2009) reported that *B. tabaci* prefer cultivated tomato plants to wild tomato plants, and their work showed that wild tomato plants released higher levels of terpenes, such as p-cymene, α-terpinene, γ-terpinene and phellandrene, which act as a repellent to *B. tabaci*. Besides monoterpenes, the antennae of the whitefly are also able to detect certain sesquiterpenes, such as zinziberene and curcumene even in low concentrations. Olson et al. (2009) reported that *Spodoptera exigua* larvae preferred a higher level of nitrogen receiving induced cotton plants during their growth to recommended level of nitrogen-treated plant, because of lower amounts of terpene volatiles (e.g., ocimene, linalool, indole) emitted from the higher level of nitrogen receiving cotton plants in compare to recommended level of nitrogen receiving plant. These results support the conclusion that nitrogen plays a role in the release of terpenes from tomato plants, which affect host preference of *B. tabaci*.

This experiment provide a valuable message that high levels of nitrogen hampers the defenses of tomato plant against *B. tabaci* by decreasing the quantity of different volatile organic compounds, including β-pinene, (+)-4-carene, α-terpinene, p-cymene, β-phellandrene, α-copaene, β-caryophyllene, and α-humulene. Further study is needed to evaluate each of these volatiles for its effect on the behavior of *B. tabaci* to find out which has the most adverse effect on *B. tabaci* as a repellent or an attractant. This finding can be used in future to identify *B. tabaci* repellents and attractants that could be used as tools of IPM of *B. tabaci* and other whitefly pests.

## Author Contributions

Conceived and designed the experiments: MI, AH, T-XL. Performed the experiments: MI, AH, YZ, and Z-FZ. Analyzed the data: MI, AH, and YZ. Contributed reagents/materials/analysis tools: MI, AH, YZ, and Z-FZ. Wrote the paper: MI, AH, and T-XL.

## Conflict of Interest Statement

The authors declare that the research was conducted in the absence of any commercial or financial relationships that could be construed as a potential conflict of interest. The reviewer SG and handling Editor declared their shared affiliation, and the handling Editor states that the process nevertheless met the standards of a fair and objective review.

## References

[B1] Adams R. P. (2009). *Identification of Essential Oil Components by Gas Chromatography/Mass Spectrometry*. Carol Stream, IL: Allured Books.

[B2] Addesso K. A. McAuslane H. J. (2009). Pepper weevils attraction to volatiles from host and nonhost plants. *Chem. Ecol.* 38 216–224. 10.1603/022.038.0127 19791617

[B3] Akol A. M. Njagi P. G. N. Sithanantham S. Muke J. M. (2003). Effects of two neem insecticide formulations on the attractiveness, acceptability and suitability of diamondback moth larvae to the parasitoid, *Diadegma mollipla* (Holmgren) (Hym., Ichneumonidae). *J. Appl. Entomol.* 127 325–331. 10.1046/j.1439-0418.2003.00771.x

[B4] Babushok V. I. Zenkevich I. G. (2008). Retention indices for most frequently reported essential oil compounds in GC. *Chromatographia* 69 257–269. 10.1365/s10337-008-0872-3

[B5] Bari M. N. Sarder M. A. (1998). Control strategy of aphid with predator, *Menochilus sexmaculatus* (F.) and insecticides. *Bangladesh J. Entomol.* 8 21–29.

[B6] Bentz J. A. Reeves J. I. I. I. Barbosa P. Francis B. (1995a). Within-plant variation in nitrogen and sugar content of poinsettia and its effects on the oviposition pattern, survival, and development to *Bemisia argentifolii*. *Environ. Entomol.* 24 271–277.

[B7] Bentz J. A. Reeves J. I. I. I. Barbosa P. Francis B. (1995b). Nitrogen fertilizer effect on selection, acceptance and suitability of *Euphorbia pulcherrima* as a host plant to *Bemisia tabaci*. *Environ. Entomol.* 24 40–45. 10.1093/ee/24.1.40

[B8] Berenbaum M. R. (1995). The chemistry of defense: theory and practice (1995). *Proc. Natl. Acad. Sci. U.S.A.* 92 2–8. 10.1073/pnas.92.1.2 7816816 PMC42807

[B9] Bernays E. A. (1990). *Insect-Plant Interactions VII.* Florida: CRC Press.

[B10] Bernays E. A. Chapman R. F. (1994a). “Chemicals in plants,” in *Host-Plant Selection by Phytophagous Insects*, eds Bernays E. A. Chapman R. F. (New York, NY: Chapman & Hall), 14–60.

[B11] Bernays E. A. Chapman R. F. (1994b). *Host-Plant Selection by Phytophagous Insects.* New York, NY: Chapman and Hall 10.1007/b102508

[B12] Bhowmik D. Kumar K. P. S. Paswan S. Srivastava S. (2012). Tomato-a natural medicine and its health benefits. *J. Pharmac. Phytochem.* 1 33–43.

[B13] Bleeker P. M. Diergaarde P. J. Ament K. Guerra J. Weidner M. Schutz S. (2009). The role of specific tomato volatiles in tomato–whitefly interaction. *Plant Physiol.* 151 925–935. 10.1104/pp.109.142661 19692533 PMC2754627

[B14] Blight M. M. Le Metayer M. Delegue M. H. P. Pickett J. A. Marion-Poll F. Wadhams L. J. (1997). Identification of floral volatiles involved in recognition of oilseed rape flowers, *Brassica napus* by honeybees, *Apis mellifera*. *J. Chem. Ecol.* 23 1715–1727. 10.1023/B:JOEC.0000006446.21160.c1

[B15] Chau A. Heinz M. Davies F. T. Jr. (2005). Influences of fertilization on *Aphis gossypii* and insecticide usage. *J. Appl. Entomol.* 129 89–97. 10.1111/j.1439-0418.2005.00943.x

[B16] Chen Y. Olson D. M. Ruberson J. R. (2010). Effects of nitrogen fertilization on tritrophic interactions. *Arthropod Plant Interact.* 4 81–94. 10.1111/1744-7917.12123

[B17] Chen Y. Ruberson J. R. Olson D. M. (2008). Nitrogen fertilization rate affects larval performance and feeding, and oviposition preference of the beet armyworm, *Spodoptera exigua*, on cotton. *Entomol. Exp. Appl.* 126 244–255. 10.1111/j.1570-7458.2007.00662.x

[B18] Cook S. M. Khan Z. R. Pickett J. A. (2007a). The use of push-pull strategies in integrated pest management. *Annu. Rev. Entomol.* 52 375–400. 16968206 10.1146/annurev.ento.52.110405.091407

[B19] Cook S. M. Rasmussen H. B. Birkett M. A. Murray D. A. Pye B. J. Watts N. P. (2007b). Behavioural and chemical ecology underlying the success of turnip rape (*Brassica napus*) from the pollen beetle (*Meligethes aeneus*). *Arthropod Plant Interact.* 1 57–67. 10.1007/s11829-007-9004-5

[B20] Da Cruz B. P. De Castro E. M. Cardoso M. D. G. De Souza K. F. Machado S. M. F. Pompeu P. V. (2014). Comparison of leaf anatomy and essential oils from *Drimys brasiliensis* Miers in a montane cloud forest in Itamonte, MG, Brazil. *Bot. Stud.* 55 41 10.1186/s40529-014-0041-y PMC5432844 28510932

[B21] Das P. Raina R. Prasad A. Sen A. (2007). Electroantennogram responses of the potato tuber moth, *Phthorimaea operculella* (Lepidoptera; Gelichiidae) to plant volatiles. *J. Biosci.* 32 339–349. 10.1007/s12038-007-0033-0 17435325

[B22] Dicke M. Baldwin I. T. (2010). The evolutionary context for herbivore-induced plant volatiles: beyond the ‘cry for help’. *Trends Plant Sci.* 15 167–175. 10.1016/j.tplants.2009.12.002 20047849

[B23] Duffey S. S. Stout M. J. (1996). Antinutritive and toxic components of plant defense against insects. *Arch. Insect Biochem. Physiol.* 32 3–37. 10.1002/(SICI)1520-6327(1996)32:1<3::AID-ARCH2>3.0.CO;2-1

[B24] Egigu M. C. Ibrahim M. A. Riikonen J. Yahya A. Holopainen T. Tiitto R. J. (2014). Effects of rising temperature on secondary compounds of yeheb (*Cordeauxia edulis* Hemsley). *Am. J. Plant. Sci.* 5 517–527. 10.4236/ajps.2014.55066

[B25] Engel E. Baty C. Le Corre D. Souchon I. Martin N. (2002). Flavor active compounds potentially implicated in cooked cauliflower acceptance. *J. Agric. Food Chem.* 50 6459–6467. 10.1021/jf025579u 12381134

[B26] Engström L. Stenberg M. Aronsson H. Linden B. (2011). Reducing nitrate leaching after winter oilseed rape and peas in mild and cold winters. *Agron. Sustain. Dev.* 31 337–347. 10.1051/agro/2010035

[B27] Fang Y. Jiao X. Xie W. Wang S. Wu Q. Shi X. (2013). Tomato yellow leaf curl virus alters the host preferences of its vector *Bemisia tabaci*. *Sci. Rep.* 3:2876 10.1038/srep02876 PMC3791452 24096821

[B28] Finch S. (1980). “Chemical attraction of plant-feeding insects to plants,” in *Applied Biology*, ed. Coaker T. H. (New York, NY: Academic Press), 67–143.

[B29] Franco C. R. P. Alves P. B. Andrade D. M. De Jesus H. C. R. Silva E. J. S. Santos E. A. B. (2011). Essential oil composition and variability in *Hyptis fruticosa*. *Braz. J. Pharmacol.* 21 24–32. 10.1002/ptr.3455

[B30] Fretz T. A. (1976). Effect of photoperiod and nitrogen on the composition of foliar monoterpenes of *Juniperus horizontalis* Moench. cv *Plumosa*. *J. Am. Soc. Hortic. Sci.* 101 611–613.

[B31] Frohlich D. R. Torres J. I. Bedford I. D. Markham P. G. Brown J. K. (1999). A phylogeographical analysis of *Bemisia tabaci* species complex based on mitochondrial DNA markers. *Mol. Ecol.* 8 1683–1691. 10.1046/j.1365-294x.1999.00754.x 10583831

[B32] Germano L. D. L. Picanço M. Zanuncio J. C. Moreira M. D. Jham G. N. (2011). Hosting capacity of horticultural plants for insect pests in Brazil. *Chil. J. Agric. Res.* 71 383–389. 10.4067/S0718-58392011000300006

[B33] Gershenzon J. (1994). Metabolic costs of terpenoid accumulation in higher plants. *J. Chem. Ecol.* 20 1281–1321. 10.1007/BF02059810 24242341

[B34] Gershenzon J. Croteau R. (1991). “Terpenoids,” in *Herbivores: Their Interactions with Secondary Plant Metabolites*, eds Rosenthal G. A. Berembaum M. R. (Boca Raton, FL: CRC Press), 165–219. 10.1016/B978-0-12-597183-6.50010-3

[B35] Gonzales W. L. Ramirez C. C. Olea N. Niemeyer H. M. (2002). Host plant changes produced by the aphid *Sipha flava*: consequences for aphid feeding behavior and growth. *Entomol. Exp. Appl.* 103 107–113. 10.1046/j.1570-7458.2002.00964.x

[B36] Gouinguenè S. Turlings T. C. J. (2002). The effects of abiotic factors on induced volatile emission in corn plants. *Plant Physiol.* 129 1296–1307. 10.1104/pp.001941 12114583 PMC166523

[B37] Han P. Lavoir A. V. Bot J. L. Desneux E. A. Desneux N. (2014). Nitrogen and water availability to tomato plants triggers bottom-up effects on the leafminer, *Tuta absoluta*. *Sci. Rep.* 4:4455 10.1038/srep04455 PMC5380111 24675796

[B38] Hasanuzzaman A. T. M. Islam M. N. Zhang Y. Zhang C.-Y. Liu T.-X. (2016). Leaf morphological characters can be a factor for intra-varietal preference of whitefly *Bemisia tabaci* (Hemiptera:Aleyrodidae) among eggplant varieties. *PLoS ONE* 11:e0153880 10.1371/journal.pone.0153880 PMC4833341 27081849

[B39] Herms D. A. Mattson W. J. (1992). The dilemma of plants: to grow or defend. *Q. Rev. Biol.* 67 283–335. 10.1086/417659

[B40] Hognadottir A. Rouseff R. L. (2003). Identification of aroma active compounds in orange essence oil using gas chromatography-olfactometry and gas chromatography-mass spectrometry. *J. Chromatogr. A* 998 201–211. 10.1016/S0021-9673(03)00524-7 12862384

[B41] Inbar M. Gerling D. (2008). Plant-mediated interactions between whiteflies, herbivores, and natural enemies. *Annu. Rev. Entomol.* 53 431–448. 10.1146/annurev.ento.53.032107.122456 17877454

[B42] Jang S. Hamayun M. Sohn E. Shin D. Kim K. Lee B. M. (2008). Effect of elevated nitrogen levels on endogenous gibberellin and jasmonic acid contents of three rice (*Oryza sativa* L.) cultivars. *J. Plant Nutr. Soil Sci.* 171 181–186. 10.1002/jpln.200625025

[B43] Jansen R. M. C. Hofstee J. W. Wildt J. Verstappen F. W. A. Bouwmeester H. J. Posthumus M. A. (2008). Health monitoring of plants by their emitter volatiles: trichome damage and cell membrane damage are detectable at greenhouse scale. *Ann. Appl. Biol.* 154 441–452. 10.1111/j.1744-7348.2008.00311.x

[B44] Jauset A. M. Yokomi R. K. Mayer R. T. Shapiro J. P. (1995). Cytology and physiology of silverleaf whitefly-induced squash silverleaf. *Exp. Appl.* 46 227–242.

[B45] Lange W. H. Bronson L. (1981). Insect pests of tomato. *Annu. Rev. Entomol.* 26 345–371. 10.1146/annurev.en.26.010181.002021

[B46] Le Quere J. L. Latrasse A. (1990). Composition of the essential oils of blackcurrant buds (*Ribes nigrum* L.). *J. Agric. Food Chem.* 38 3–10. 10.1021/jf00091a001

[B47] Lee K. D. Yang M. S. Supanjani Smith D. L. (2005). Fertilizer effect on the yield and terpene components from the flower heads of *Chrysanthemum boreale* M. (Compositae). *Agron. Sustain. Dev.* 25 205–211. 10.1051/agro:2005022

[B48] Li T. Y. Maschwitz U. (1985). The sexual behavior of whitefly *Trialeurodes vaporariorum*. *Acta Entomol. Sin.* 28 233–235.

[B49] Liu T.-X. (2007). Life history of *Eretmocerus melanoscutus* (Hymenoptera: Aphelinidae) parasitizing nymphs of *Bemisia tabaci* biotype B (Homoptera: Aleyrodidae). *Biol. Control* 42 77–85. 10.1016/j.biocontrol.2007.03.008

[B50] Locascio S. J. Clark G. A. Czizinszky A. A. Stanley C. D. Olson S. M. Rhoads F. M. (1992). *Water and Nutrient Requirements for Drip-Irrigated Vegetables in Humid Regions.* Gainesville, FL: University of Florida.

[B51] Luan J. B. Yao D. M. Zhang T. Walling L. L. Yang M. Wang Y. J. (2013). Suppression of terpenoid synthesis in plants by a virus promotes its mutualism with vectors. *Ecol. Lett.* 16 390–398. 10.1111/ele.12055 23279824

[B52] Mattson W. J. (1980). Herbivory in relation to plant nitrogen content. *Annu. Rev. Ecol. Syst.* 11 19–61. 10.1146/annurev.es.11.110180.001003

[B53] Mumm R. Dicke M. (2010). Variation in natural plant products and the attraction of bodyguards for indirect plant defense. *Can. J. Zool.* 88 628–667. 10.1139/Z10-032

[B54] Nampoothiri S. V. Venugopalan V. V. Joy B. Sreekumar M. M. Menon A. N. (2012). Comparison of essential oil composition of three ginger cultivars from sub Himalayan region. *Asia Pac. J. Trop. Biomed.* 2 S1347–S1350. 10.1016/s2221-1691(12)60414-6

[B55] Olaniyi J. O. Akanbi W. B. Adejumo T. A. Akande O. G. (2010). Growth, fruit yield and nutritional quality of tomato varieties. *Afr. J. Food Sci.* 4 398–402.

[B56] Olson D. M. Cortesero A. M. Rains G. C. Potter T. Joe Lewis W. (2009). Nitrogen and water affect direct and indirect plant systemic induced defense in cotton. *Biol. Control.* 49 239–244. 10.1016/j.biocontrol.2009.02.011

[B57] Owolabi M. S. Oladimeji M. O. Labunmi L. Singh G. Marimuthu P. Valery A. I. (2007). Composition and biological potentials of the essential oil of *Zingiber officinale* (Roscoe) from Nigeria. *Bull. Pure Appl. Sci.* 26 113–119.

[B58] Raguso R. A. Light D. M. Pickersky E. (1996). Electroantennogram responses of *Hyles lineata* (Sphingidae: Lepidoptera) to volatile compounds from *Clarkia breweri* (Onagraceae) and other moth- pollinated flowers. *J. Chem. Ecol.* 22 1735–1766. 10.1007/BF02028502 24227106

[B59] Reddy A. S. Rao V. N. (1989). Cotton whitefly (*Bemisia tabaci* Genn.) A review. *Indian J. Plant Protec.* 17 171–179.

[B60] Saad K. A. Roff M. N. M. Hallett R. H. Idris A. B. (2015). Aphid-induced defenses in chilli affect preferences of the whitefly, *Bemisia tabaci* (Hemiptera: Aleyrodidae). *Sci. Rep.* 5:13697 10.1038/srep13697 PMC4558579 26334135

[B61] Saad K. A. Roff M. N. M. Shukri M. A. M. Mirad R. Mansour S. A. A. Abuzid I. (2013). Behavioral responses of whitefly, *Bemisia tabaci* (Hemiptera: Aleyrodidae), in relation to sex and infestation status of their host plants. *Acad. J. Entomol.* 6 95–99.

[B62] Sa-Nguanpuag K. Kanlayanarat S. Srilaong V. Tanprasert K. Techavuthiporn C. (2011). Ginger (*Zingiber officinale*) oil as an antimicrobial agent for minimally processed produce: a case study in shredded green papaya. *Int. J. Agric. Biol.* 13 895–901.

[B63] Schoonhoven L. M. Vanloon J. J. A. Dicke M. (2005). *Insect-Plant Biology.* Oxford: Oxford University Press.

[B64] Sieling K. Kage H. (2010). Efficient N management using winter oilseed rape. A review. *Agron. Sustain. Dev.* 30 271–279. 10.1051/agro/2009036

[B65] Simmons A. M. Gurr G. M. (2005). Trichomes of *Lycopersicon* species and their hybrids: effects on pests and natural enemies. *Agric. For. Entomol.* 7 265–276. 10.1111/j.1461-9555.2005.00271.x

[B66] Simpson S. J. Simpson C. L. (1990). “The mechanisms of nutritional compensation by phytophagous insects,” in *Insect-Plant Interactions*, Vol. II, ed. Bernays E. A. (Boca Raton, FL: CPC Press), 111–160.

[B67] Slosser J. E. Parajulee M. N. Hendrix D. L. Henneberry T. J. Pinchak W. E. (2004). Cotton aphid (Homoptera: Aphididae) abundance in relation to cotton leaf sugars. *Environ. Entomol.* 33 690–699. 10.1603/0046-225X-33.3.690

[B68] Southwood T. R. E. (1973). “The insect/plant relationship–an evolutionary perspective,” in *Insect-plant Relationship* Vol. 6 ed. Emden F. V. (London: Symp. Royal Entomol. Soc), 3–30.

[B69] Stout M. J. Brovont R. A. Duffey S. S. (1998). Effects of nitrogen availability on expression of constitutive and inducible chemical defenses in tomato. *J. Chem. Ecol.* 24 945–963. 10.1023/A:1022350100718

[B70] Szendrei Z. Rodriguèz-Saona C. A. (2010). A meta-analysis of insect pest behavioural manipulation with plant volatiles. *Entomol. Exp. Appl.* 134 201–210. 10.1111/j.1570-7458.2009.00954.x

[B71] Toscano L. C. Boica A. L. Maruyama W. I. (2002). Non-preference of whitefly for oviposition in tomato genotypes. *Sci. Agric.* 59 677–681. 10.1590/S0103-90162002000400009

[B72] Tuomi J. Niemela P. Haukioja E. Siren S. Neuvonen S. (1994). Nutrient stress: an explanation for plant anti-herbivore responses to defoliation. *Oecologia* 61 208–210. 10.1007/BF00396762 28309413

[B73] Ukeh D. A. Birkett M. A. Pickett J. A. Bowman A. S. Mordue Luntz A. J. (2009). Repellent activity of alligator pepper, *Aframomum melegueta*, and ginger, *Zingiber officinale*, against the maize weevil, *Sitophilus zeamais*. *Phytochemistry* 70 751–758. 10.1016/j.phytochem.2009.03.012 19394981

[B74] Unsicker S. B. Kunert G. Gershenzon J. (2009). Protective perfumes: the role of vegetative volatiles in plant defense against herbivores. *Curr. Opin. Plant Biol.* 12 479–485. 10.1016/j.pbi.2009.04.001 19467919

[B75] Van Den Dool H. Kratz P. D. J. A. (1963). A generalization of the retention index system including linear temperature programmed gas-liquid partition chromatography. *J. Chromatogr.* 11 463–471. 10.1016/S0021-9673(01)80947-X 14062605

[B76] Van Wassenhove F. A. Dirinck P. J. Schamp N. M. Vulsteke G. A. (1990). Effect of nitrogen fertilizers on celery volatiles. *J. Agric. Food Chem.* 38 220–226. 10.1021/jf00091a049

[B77] Veromann E. Toome M. Kännaste A. Kaasik R. Copolovici L. Flink J. (2013). Effects of nitrogen fertilization on insect pests, their parasitoids, plant diseases and volatile organic compounds in *Brassica napus*. *Crop. Prot.* 43 79–88. 10.1016/j.cropro.2012.09.001

[B78] Wahle E. A. Masiunas J. B. (2003). Population density and nitrogen fertility effects on tomato growth and yield. *Hort. Sci.* 38 367–372.

[B79] Wang Y. T. Huang S. W. Liu R. L. Jin J. Y. (2007). Effects of nitrogen application on flavor compounds of cherry tomato fruits. *J. Plant Nutr. Soil Sci.* 170 1–8. 10.1002/jpln.200700011

[B80] Warneke C. Van der Veen C. Luxembourg S. L. de Gouw J. A. Kok A. (2001). Measurements of benzene and toluene in ambient air using proton-transfer-reaction massspectrometry: calibration, humidity dependence and field intercomparison. *Int. J. Mass Spectrom.* 207 167–182. 10.1016/S1387-3806(01)00366-9

[B81] Yang N. W. Li A. L. Wan F. H. Liu W. X. Johnson D. (2010). Effects of plant essential oils on immature and adult sweetpotato whitefly, *Bemisia tabaci* biotype B. *Crop Prot.* 29 1200–1207. 10.1016/j.cropro.2010.05.006

[B82] Ying J. Huang J. Rui-yan M. Ju-cai H. (2003). Host plant preferences of *Bemisia tabaci* Gennadius. *Insect Sci.* 10 109–114. 10.1111/j.1744-7917.2003.tb00372.x

[B83] Zaini M. R. Rawi C. S. M. Hassan A. (2013). Effect of nutrient and pre-infested brinjal, *Solanum melongena* by whitefly and aphid on population dynamics of whitefly, *Bemisia tabaci*. *Agric. For. Fish.* 2 1–10. 10.11648/j.aff.20130201.11

[B84] Zhang A. Linn C. Wright S. Prokopy R. Reissig W. Roelofs W. (1999). Identification of a new blend of apple volatiles attractive to the apple maggot, *Rhagoletis pomonella*. *J. Chem. Ecol.* 25 1221–1232. 10.1007/BF00989104 24414891

[B85] Zhang W. McAuslane H. J. Schuster D. J. (2004). Repellency of ginger oil to *Bemisia argentifolii* (Homoptera: Aleyrodidae) on tomato. *J. Econ. Entomol.* 97 1310–1318. 10.1093/jee/97.4.1310 15384342

[B86] Zheng L. X. Wu W. J. Liang G. W. Fu Y. G. (2013). 3,3-dimethyl-1-butanol, a parakairomone component to *Aleurodicus dispersus* (Hemiptera: Aleyrodidae). *Arthropod Plant Interact.* 7 423–429. 10.1007/s11829-013-9258-z

[B87] Zotarelli L. Scholberg J. M. Dukes M. D. Carpena R. M. (2007). Monitoring of nitrate leaching in sandy soils: comparison of three methods. *J. Environ. Qual.* 36 953–962. 10.2134/jeq2006.0292 17526874

[B88] Zu Dohna H. (2006). The distribution of eggs per host in a herbivorous insect-intersection of oviposition, dispersal and population dynamics. *J. Anim. Ecol.* 75 387–395. 10.1111/j.1365-2656.2006.01059.x 16637992

