# Supplementary figures and images for: High Level of Nitrogen Makes Tomato Plants Releasing Less Volatiles and Attracting More Bemisia tabaci (Hemiptera: Aleyrodidae)
Source: Front Plant Sci. 2017 Mar 31;8:466. doi: 10.3389/fpls.2017.00466 (PMC5374211; doi:10.3389/fpls.2017.00466)

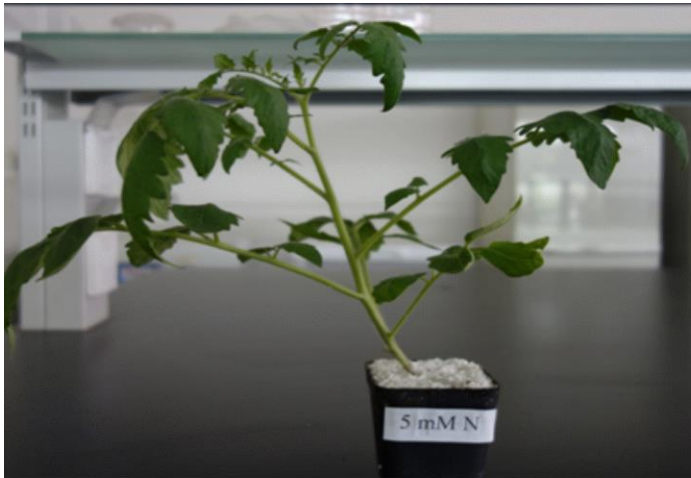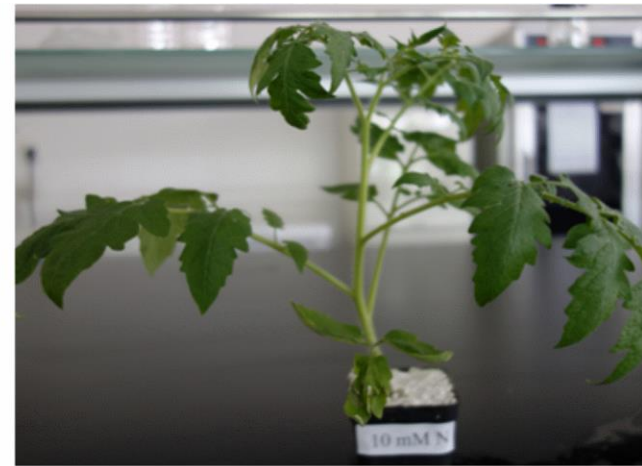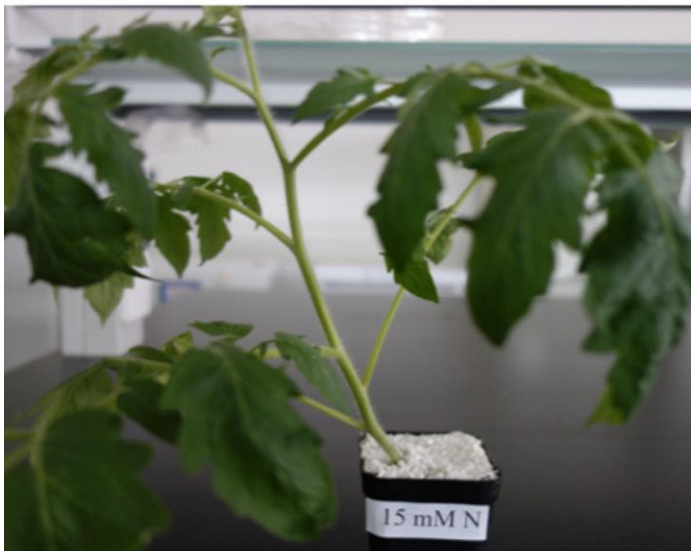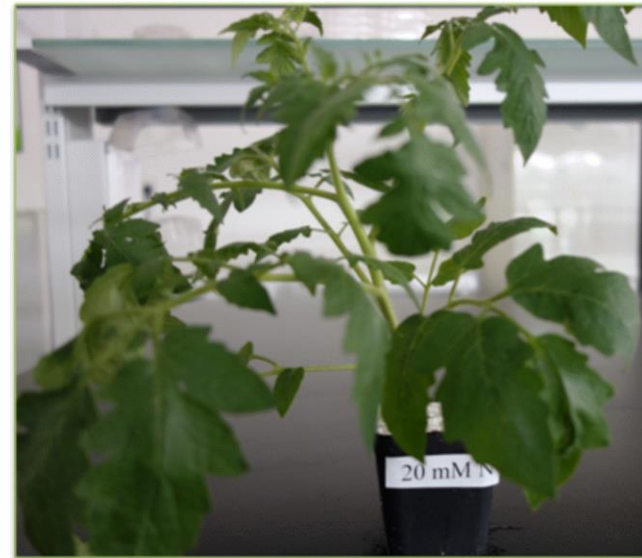

**Figure S1. Different levels of nitrogen treated tomato plants.**

Supplement: Supplementary file 1 [file Image_1.PDF]

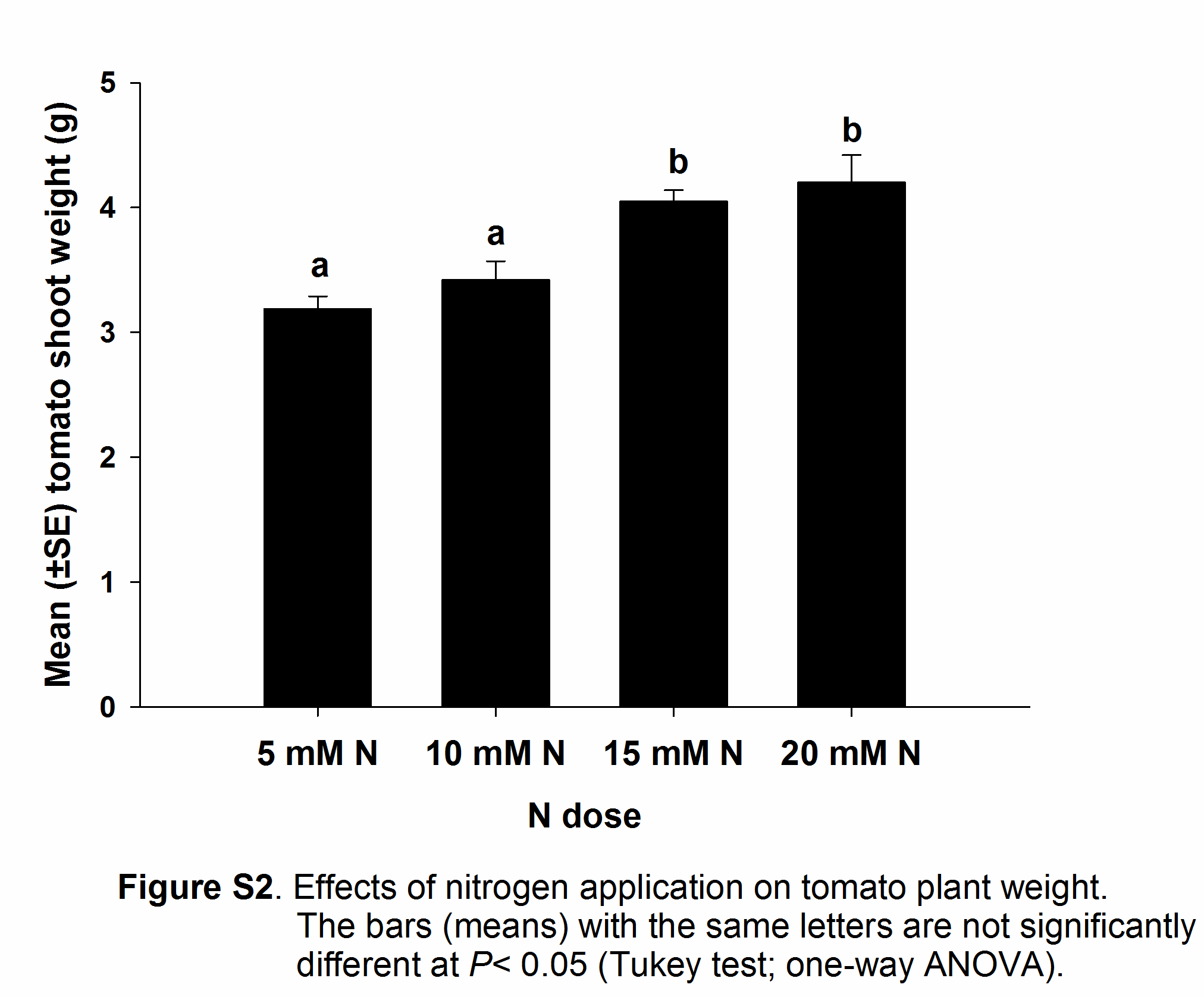

Supplement: Supplementary file 2 [file Image_2.TIF]
